# Circulating antibodies against age-modified proteins in patients with coronary atherosclerosis

**DOI:** 10.1038/s41598-020-73877-5

**Published:** 2020-10-13

**Authors:** Edina Korça, Veronika Piskovatska, Jochen Börgermann, Alexander Navarrete Santos, Andreas Simm

**Affiliations:** 1 Department of Cardiothoracic Surgery, University Hospital Halle (Saale), Martin-Luther University Halle-Wittenberg, Halle, Germany; 2 Center for Medical Basic Research, Martin-Luther University Halle-Wittenberg, Halle, Germany; 3 Klinik für Herzchirurgie, Mitteldeutsches Herzzentrum, Ernst-Grube-Str. 40, 06120 Halle (Saale), Germany; 4 Present Address: Herzzentrum Duisburg, Duisburg, Germany

**Keywords:** Biomarkers, Diseases, Risk factors

## Abstract

Advanced glycation endproducts (AGEs) are formed in a series of non-enzymatic reactions between reducing sugars and the amino groups of proteins and accumulate during aging, diabetes mellitus, chronic kidney disease and other chronic diseases. Accumulation of AGE-modifications alters protein structure and function, transforming these molecules into potential targets of the immune system, presumably triggering the production of autoantibodies against AGEs. In this study, we detected autoantibodies against AGE-modified proteins with ELISA in plasma samples of 91 patients with documented coronary artery disease (CAD), who underwent coronary artery bypass grafting (CABG) surgery. Patients with high levels of autoantibodies had a higher body mass index (BMI 28.6 vs 27.1 kg/m^2^; p = 0.046), were more likely to suffer from chronic obstructive pulmonary disease (COPD 30% vs 9.8%; p = 0.018), and more likely to need dialysis after the surgery (10% vs 0%; p = 0.037). Our findings show a weak link between the levels of autoantibodies against AGEs and diabetes mellitus (DM 44% vs 24.4%; p = 0.05). In a small subpopulation of patients, antibodies against native bovine serum albumin (BSA) were detected. A growing body of research explores the potential role of antibodies against AGE-modified proteins in pathogenesis of different chronic diseases; our data confirms the presence of AGE-autoantibodies in patients with CAD and that in parallel to the AGEs themselves, they may have a potential role in concomitant clinical conditions in patients undergoing CABG surgery. Further research is necessary to verify the molecular role of these antibodies in different pathological conditions.

## Introduction

Advanced glycation endproducts (AGEs) are formed after a series of non-enzymatic reactions between reducing sugars (glucose, fructose, ribose) or dicarbonyls with proteins, peptides, lipids or nucleic acids. In the first step, an unstable Schiff’s base is formed which undergoes rearrangements to form Amadori products, followed by a series of complex reactions to finally form AGEs. The reaction was first described by Louis Camille Maillard and is thus often referred to as the “Maillard reaction”^1^.

AGEs are implicated in a number of age-related diseases and conditions such as diabetes mellitus (DM) and its complications, Alzheimer's disease, vascular dementia, atherosclerosis, hypertension and chronic kidney disease (CKD)^1–4^.

Structural alterations due to glycation can form new epitopes on proteins. These epitopes are potentially recognized as unfamiliar by the immune system and thus trigger an immune response leading to the production of autoantibodies against AGE-modified proteins (Anti-AGE-Abs). Research on this topic is scarce and the physiological role of these autoantibodies is still unclear. However, their existence has been confirmed by several studies, showing that these Anti-AGE-Abs are present in healthy subjects as well as in different diseases. Most of the available data show that Anti-AGE-Abs are more abundant in pathological conditions such as diabetes, rheumatoid arthritis (RA) or lupus when compared to healthy subjects^5–7^.

However, the presence of Anti-AGE-Ab has been studied mainly in patients with diabetes. Ashraf and colleagues detected the presence of autoantibodies against glycated histone H2A in patients with DM. These autoantibodies specifically bind to carboxymethyllysine (CML) and pentosidine, the most abundant AGEs^5^. Ashraf et al. also detected autoantibodies against AGE-modified-DNA in patients with DM and found out that with longer disease duration the Anti-AGE-Ab level rises^8^. Additionally, autoantibodies against glycated low-density-lipoprotein (LDL) have been detected in patients with DM^5^. Whereas DM is a clear risk factor for cardiovascular diseases, Type 2 Diabetes seems to be protective for chronic obstructive pulmonary disease (COPD)^9^. As all three diseases share inflammation as a common pathophysiological mechanism, it would be of interest if autoantibodies against AGEs are associated with COPD as well.

Several research groups have also detected Anti-AGE-Ab in patients with chronic conditions other than DM. The presence of AGE-modified IgG was investigated in patients with rheumatoid arthritis (RA). Autoantibodies were shown to have preferential binding to glycated IgG in comparison to native IgG^7,10^. Alam et al. detected autoantibodies against deoxyribose-modified-H2A in patients with systemic lupus erythematosus (SLE) and suggested possible cross-reaction of these autoantibodies with native DNA^6^.

Not only intrinsic AGEs can be immunogenic, but also AGE-modifications on food proteins are thought to play an important role in the development of IgE-mediated allergies^11,12^. It has been suggested that dietary AGEs have a role in the onset and progression of chronic diseases, and have a considerable role in overall modifications of human proteome^13,14^.

Independent studies indicate that certain proteins might be more readily recognized as neoallergens by the immune system when introduced as Maillard reaction products^15^. Experiments in several different cell-culture models demonstrated that exposure to AGE-modified food allergens, such as ovalbumin or Ara-h 1 protein leads to increased expression of RAGE, activation of the transcription factor NF-κB^16^ and modulated secretion of IL-8, affecting proliferation of the cells^17^ compared to stimulation with unmodified proteins. Immunogenicity of AGE-modified proteins is mediated via interaction with RAGE and scavenger receptor class A type I and II^16,18^. AGEs can mimic biological actions of specific molecules like alarmins (i.e. S100 protein and high molecular group box 1), stemming from damaged or dead tissues. Binding to the RAGE, both alarmins and AGEs can affect innate and adaptive immune responses^19^. Modern industrialized diets, rich in processed foods, AGEs and sugars could result in immunological misinterpretation of a threat from dietary proteins, promoting the onset of food allergy^20,21^.

Since glycation and production of Anti-AGE-Ab is happening ubiquitously, their role in pathogenesis of diseases should be addressed more carefully. Having in mind that AGEs are supposed to play a role in atherosclerosis^22,23^, we explored potential connections between AGEs, sRAGE and anti-AGE-Abs in patients with CAD undergoing CABG. Our aim was to determine whether there is a correlation between the amount of autoantibodies, concomitant diseases, clinical parameters and outcome of the patients with CAD.

## Patients and methods

### Study population

91 patients with CAD, documented by angiography and selected for further CABG surgery, were enrolled into the study in 2 clinical centers: Department of Cardiothoracic Surgery of the University Hospital of Halle and the Heart Centre Coswig. The study design was approved by the local ethics committee, the ethical committee of the Medical Faculty of the Martin-Luther-University Halle-Wittenberg; all patients signed an informed consent before entering the study (ClinicalTrials.gov number, NCT00999089).All experiments were performed in accordance with the guidelines.

All participants underwent a standard clinical examination. Patients were considered hypertensive if they presented a blood pressure higher than 140/90 mmHg or received antihypertensive medication. Diabetes was diagnosed according to the criteria of the American Diabetes Association. European System for Cardiac Operative Risk Evaluation (euroSCORE MS Excel interactive calculator) was used to calculate the operative mortality rate following heart surgery (https://www.euroscore.org/calculators.htm). The functional class of heart failure was assessed according to New York Heart Association (NYHA) classification.

Blood samples were collected in heparin-containing tubes at nine different time points: before the operation (T1), after induction of narcosis (T2), after sternotomy (T3), after opening aortic clamps, beginning of reperfusion (T4), at the end of the operation (T5), 5 h postoperatively (T6), 24 h postoperatively (T7), 48 h postoperatively (T8) and 8 days after the operation (T9). Samples were centrifuged for plasma separation, specimens were stored at − 80 °C until further analysis.

Plasma specimens from T1 of the 91 patients were used for enzyme-linked immunosorbent assay (ELISA) measurements of Anti-AGE-Ab. Eight patients were selected randomly for measurement of Anti-AGE-Ab-kinetics during all 9 time-points.

### Preparation of AGE-modified BSA

AGE-modified BSA was prepared under sterile conditions by incubating native BSA (Thermo Fisher Scientific) with 50 mM ribose (Sigma) in 50 mM potassium phosphate buffer (pH 7.3) at 50 °C for 40 days. The presence of AGE-modifications (pentosidine, methylglyoxal-derived hydroimidazolone 1 (MG-H1) and CML) in ribose-modified BSA (R-BSA) was confirmed by immunoblotting. Self-made polyclonal antibodies against CML, produced in rabbit, mouse monoclonal anti-MG-H1 antibodies (Cell Biolabs) and mouse monoclonal antibodies against pentosidine (Biologo) were used.

### Detection of anti-AGE autoantibodies

A direct binding ELISA was established to detect immunoglobulin G (IgG) antibodies against AGEs. High binding, flat bottom, transparent ELISA plates (Greiner Bio) were coated with 100 µl (20 µg/ml) of native BSA (Albumin Fraction V, IgG-free, Gibco) or ribose-modified BSA (R-BSA) in antigen coating buffer (Na_2_CO_3_/NaHCO_3_ pH 9.6 + 0.1% Sodium Azide). The plates were incubated overnight at 4 °C. Next day, the plates were washed five times for one minute with 200 µl Tris-buffered saline (TBS) supplemented with 0.1% Tween-20 (TBS-T) and blocked with 200 µl of 5% BSA (Albumin Fraction V, AppliChem Panreac) in TBS for 2 h at room temperature (RT) on a plate shaker. Then, the plates were washed again five times with TBS-T and 100 µl of plasma diluted 1:50 in TBS was added to each well. Anti-CML antibody (polyclonal rabbit, in-house produced) served as positive control, and TBS served as negative control. Plates were incubated for 90 min at RT and then overnight at 4 °C on a plate shaker. After incubation, plates were washed five times with TBS-T. HRP-conjugated, Anti-IgG antibody (Rabbit Anti-Human IgG, H + L, Abcam) was added to the wells, previously incubated with plasma from the patients. Positive control wells were treated with anti-rabbit HRP-conjugated antibody (Donkey Anti-Rabbit IgG, H + L, Dianova). After 1-h incubation at RT on a plate shaker, wells were washed with TBS-T.

50 µl of developing solution (1-Step Ultra TMB-ELISA, Thermo Fisher Scientific) was added to each well and plates were incubated for 20 min in the dark. To stop the reaction, 50 µl of 2 M H_2_SO_4_ was added to each well. Absorbance was measured with spectrophotometer (FLUOstar BMG, Optima), at 450 nm wavelength.

The intra-assay coefficient of variation (CV) was 4% and inter-assay CV was 9%. Half of the plate coated with BSA served as control. Each sample was assayed in duplicate and the results were calculated as an average of two measurements. Results are presented as the difference between absorbance of test well and the BSA control well.

### Measurement of the advanced glycation index (AGI) and sRAGE in plasma

AGE-specific fluorescence of the plasma was determined according to Sampathkumar et al.^24^. An AGI is a 5-point linear regression estimate; therefore it characterizes the AGE-content/fluorescence of the plasma more accurately than the single-point measurements. Levels of plasma sRAGE were measured and already published as described in Ref.^25^ using a commercially available human RAGE Quantikine ELISA kit (R&D systems) according to the protocol recommended by the manufacturer.

### Detection of anti-BSA antibodies in plasma of patients and evaluation of their specificity

According to our results from ELISA BSA control wells we prepared a pool from plasma samples of four patients with high reactivity against native BSA. Immunodetection was performed according to the protocol of Mogues et al.^26^ with a few modifications. To prove that the reactivity stems from high levels of specific antibodies against BSA (anti-BSA-Ab), 1 µg of BSA (Albumin Fraction V, IgG-free, Gibco) and 1 µg of HSA (Sigma Aldrich A5843), was blotted onto 0.2 µm nitrocellulose membrane using Minifold I Slot-blot system (Whatman). Protein loading was controlled with REVERT total protein stain from Li-COR. Membranes were blocked with 1% HSA in PBS for 2 h at RT, and washed in PBS-T (Tween-20 0.1%). The plasma pool was diluted 1:200 in PBS added to the membranes and incubated overnight at 4 °C. Then the membranes were washed and incubated with a secondary anti-human IgG antibody (Goat Anti-Human IgG (H + L), IRDye 800CW, Li-COR) for 1 h at RT, washed again and analyzed via Odyssey imaging system (Li-COR). Signal from IgG detection was standardized towards Revert total protein stain from Li-COR.

### Removal of anti-BSA antibodies from plasma samples

In order to remove anti-BSA-Ab from the plasma, preconditioning with immobilized BSA was used. The anti-BSA-Ab were removed from the plasma pool using streptavidin magnetic beads (Thermo Fisher Scientific) conjugated to biotinylated BSA (Thermo Fisher Scientific). 10 µg of biotinylated BSA in 100 µl nuclease-free water (Promega) were added to 50 µl of streptavidin magnetic beads and incubated for 2 h at RT on rotator. After incubation, the supernatant was removed on the magnet and beads were washed 3 times with PBS. 50 µl of plasma was incubated with obtained beads, conjugated to BSA overnight at 4 °C on the rotator. After the incubation, plasma was removed and used for further immunodetection as described in the protocol above. Two aliquots of plasma were used as a potential source of anti-BSA antibodies, only one aliquot was preconditioned with immobilized BSA.

### Statistical analysis

Patients were divided into two groups according to the levels of Anti-AGE-Ab—the mean value of Anti-AGE-Abs served as a cut-off threshold for division. Data are presented as the mean ± standard error of the mean (SEM). For continuous variables, differences between two groups were evaluated with an unpaired 2-sided T-test. For categorical variables, differences between two groups were analyzed with the χ^2^ test using Fisher's exact test. A p-value p < 0.05 was considered as significant.

## Results

The study population included 91 patients, with a mean age of 66.5 ± 0.8 years. The mean body mass index (BMI) was 27.9 ± 0.4. Females comprised 26% of the study participants. About one-third of the patients presented with diabetes, 21% had chronic obstructive pulmonary disease (COPD) and 5.5% preoperative renal failure. The mean New York Heart Association (NYHA) class was 2.2 ± 0.1. Basic characteristics of the participants are shown in Table 1.

**Table 1:** Characteristics of the included patients (n = 91).

Parameter	
Age, years, mean ± SEM	66.5 ± 0.8
Women, %	26.4
BMI, mean ± SEM	27.9 ± 0.4
Blood pressure systolic, mmHg, mean ± SEM	127.5 ± 1.8
Blood pressure diastolic, mmHg, mean ± SEM	74.5 ± 1
Myocardial infarction, %	58.2
Diabetes, %	35.2
Hyperlipidemia, %	94.5
Smokers, % (active and former)	59.3
COPD, %	20.9
Renal failure, %	5.5
NYHA functional class, mean ± SEM	2.2 ± 0.1
euroSCORE risk, mean ± SEM	4 ± 0.2

*SEM* standard error of mean, *BMI* body mass index, *COPD* chronic obstructive pulmonary disease, *NYHA* New York Heart Association.

Patients were divided into two groups (patients with low Anti-AGE-Abs and patients with high Anti-AGE-Abs) according to the levels of Anti-AGE-Abs, the mean value of Anti-AGE-Abs served as a cut-off threshold for division. Group 1 involved 41 patients with low Anti-AGE-Ab levels 0.01 ± 0.06 a.u., and group 2 involved 50 patients with a high Anti-AGE-Ab levels 0.5 ± 0.02 a.u.

We evaluated the correlation of Anti-AGE-Ab level with the preoperative and postoperative available data (Tables 2, 3). The mean BMI of group 1 was 27.08 ± 0.52 kg/m^2^ and that of group 2 was 28.62 ± 0.54 kg/m^2^. Patients from group 2 with more Anti-AGE-Ab had a significantly higher BMI when compared to patients of group 1 with fewer Anti-AGE-Ab (p = 0.046). From all 91 participants, 19 had COPD. Both χ^2^ test and Fisher's exact test showed a significant correlation (p = 0.018; p = 0.02) between COPD and higher amounts of autoantibodies against AGEs. In contrast to published data regarding diabetes and Anti-AGE-Ab, only a trend towards a positive correlation (p = 0.05 in the χ^2^ test; p = 0.08 with Fisher's exact test) was observed (Table 2).

**Table 2:** Correlation of Anti-AGE-Ab levels with preoperative characteristics of patients.

	Low Anti-AGE-Ab level (n = 41)	High Anti-AGE-Ab level (n = 50)	p-value		
			T-test	χ^2^ test	Fisher's exact test
Anti-AGE-Abs, a.u., mean ± SEM	0.01 ± 0.06	0.5 ± 0.02			
BMI, mean ± SEM	27.08 ± 0.52	28.62 ± 0.54	**0.04**		
Diabetes, n	10	22		**0.05**	0.08
COPD, n	4	15		**0.02**	**0.02**
Kidney failure	2	3		0.81	1.00
NYHA functional class, mean ± SEM	2.15 ± 0.15	2.22 ± 0.13		0.71	
PAOD, n	2	8		0.09	0.10
Unstable angina pectoris	2	9		0.06	0.10
euroSCORE risk, mean ± SEM	3.71 ± 0.34	4.18 ± 0.32	0.31		
Blood pressure systolic, mmHg, mean ± SEM	125.5 ± 2.8	129.1 ± 2.4	0.3		
Blood pressure diastolic, mmHg, mean ± SEM	74.2 ± 1.6	74.7 ± 1.2	0.8		

Significant p-values below 0.05 are shown in bold.

*Anti-AGE-Ab* autoantibodies against AGEs, *SEM* standard error of the mean, *BMI* body mass index, *COPD* chronic obstructive pulmonary disease, *NYHA* New York Heart Association, *PAOD* peripheral arterial occlusive disease.

**Table 3:** Correlation of Anti-AGE-Ab levels with patient’s outcome.

	Low Anti-AGE-Ab level (n = 41)	High Anti-AGE-Ab level (n = 50)	p-value	
			χ^2^ test	Fisher's exact test
Anti-AGE-Ab, mean ± SEM	0.01 ± 0.06	0.5 ± 0.02		
Postoperative dialysis, n	0	5	**0.04**	0.07
ICU > 48 h, n	5	11	0.22	0.27
30 days mortality, n	1	2	0.67	1.00

Significant p-value below 0.05 is shown in bold.

*Anti-AGE-Ab* autoantibodies against AGEs, *SEM* standard error of the mean, *ICU* intensive care unit.

We also analyzed the correlation of autoantibody level with some postoperative parameters. Our analysis do not show any correlation between Anti-AGE-Ab level and postoperative stay in intensive care unit (ICU) or 30-day mortality rate (Table 3). Five patients of group 2 needed dialysis after the surgery, but none of group 1. However, because of the small sample size, the results are significant only according to χ^2^ test (p = 0.04), but not Fisher's exact test (p = 0.07). The patients who underwent dialysis were dialyzed on average for 15 days.

**Figure 1:**
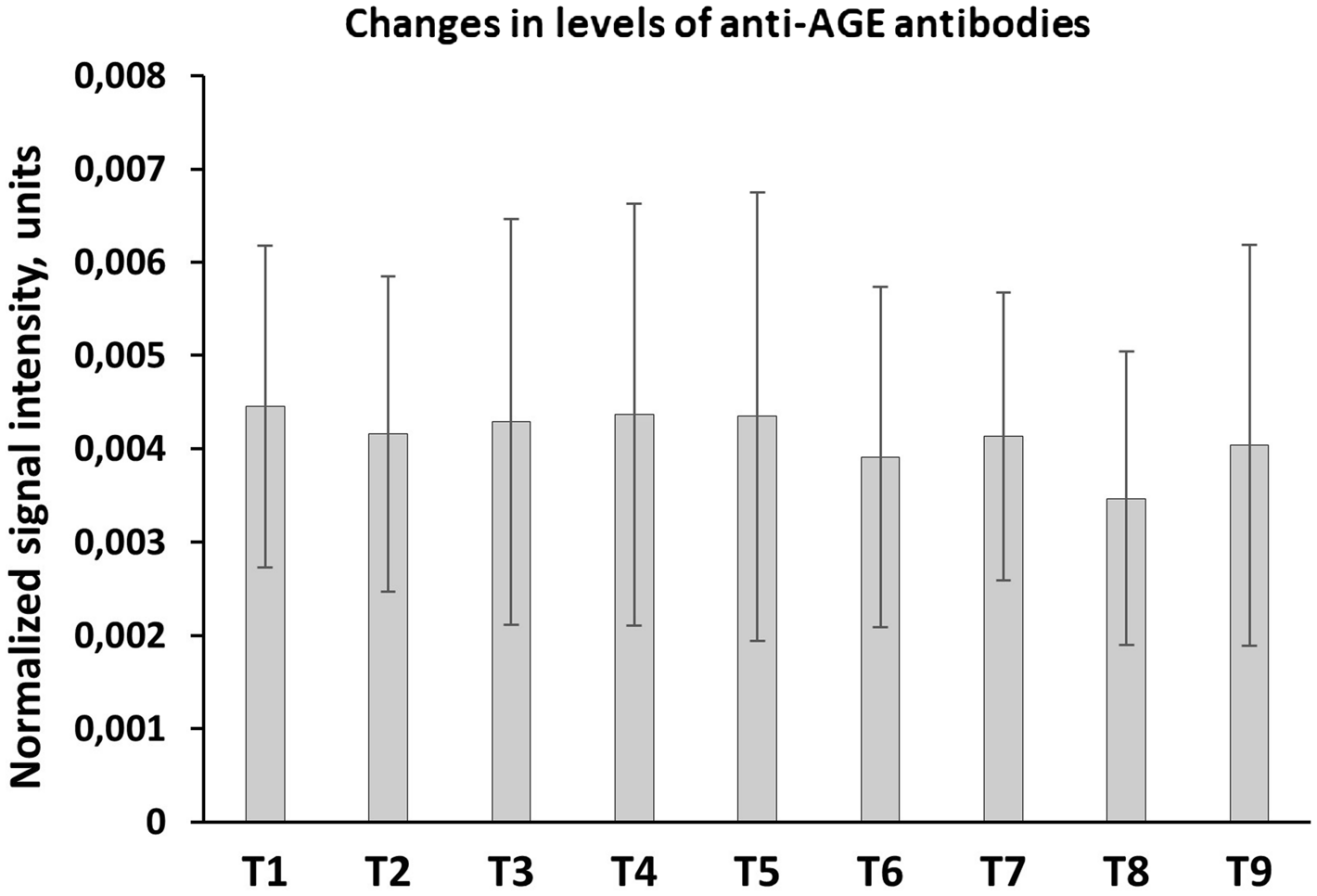
Anti-AGE-Ab kinetics over a period of 8 days after CABG surgery, mean ± SD (n = 8), plasma samples were collected before the operation (T1); after induction of narcosis (T2); after sternotomy (T3); after opening aortic clamps, beginning of reperfusion (T4); at the end of the operation (T5); 5 h postoperatively (T6); 24 h postoperatively (T7); 48 h postoperatively (T8) and 8 days after the operation (T9). Data is presented as signal intensity normalized to protein concentration (mean ± SD, n = 8). Plasma protein concentration was determined using the BCA Protein Assay Kit (Thermo Fischer Scientific).

We selected eight patients for kinetics measurement of Anti-AGE-Ab level during all nine available time-points. Results were normalized to protein concentration, due to possible blood loss and fluid replacement therapy as a consequence of surgery. The Anti-AGE-Ab levels during and up to 8 days postoperatively did not change significantly (Fig. 1).

Against expectation, the measured plasma AGE-specific fluorescence and levels of plasma sRAGE did not correlate with the amount of Anti-AGE-Abs. (Supplementary Fig. 1).

A small subpopulation of patients had detectable levels of anti-BSA-Ab leading to negative ELISA results. Immunoblotting with plasma pools from these patients showed the specificity of these IgGs towards BSA and not HSA (Supplementary Fig. 2). As part of the study we also established a method to remove anti-BSA-Ab from human plasma. This was done by incubating the plasma pools with streptavidin-magnetic beads, conjugated to biotinylated BSA (Supplementary Fig. 3). Further use of these plasma pools for immunodetection of native BSA confirmed removal of anti-BSA-Abs. Subsequent ELISA with the plasma pools depleted of anti-BSA-Abs showed no more negative results. The final Anti-AGE-Ab level of the plasma pools was under the threshold set as a cut off for low Anti-AGE-Ab levels, so values remained within the same group.

## Discussion

In pathological conditions, the overproduction of AGEs could lead to an increased production of Anti-AGE-Ab with formation of immune complexes. Existing in circulation or being deposited in tissues, immune complexes can play a role in functional deterioration of organs and tissues, acting as stimulus to enhance inflammation and fibrosis^27^. AGE-modification of LDL is supposed to play an important role in the pathogenesis of atherosclerosis. Activation of Fc receptors instead of scavenger receptors from AGE-LDL-containing immune complexes might be a cause of impaired LDL metabolism leading to enhanced formation of foam cells in the atherosclerotic plaque^5^. High level of AGE-modified LDL, as a compound of circulating immune complexes, was shown to be a predictor of macroalbuminuria in patients with type 2 DM^28^. Anti-AGE autoantibodies may cross-react with normal proteins of the body, inducing chronic inflammatory and autoimmune diseases such as systemic lupus erythematosus or rheumatoid arthritis^29^.

**Figure 2:**
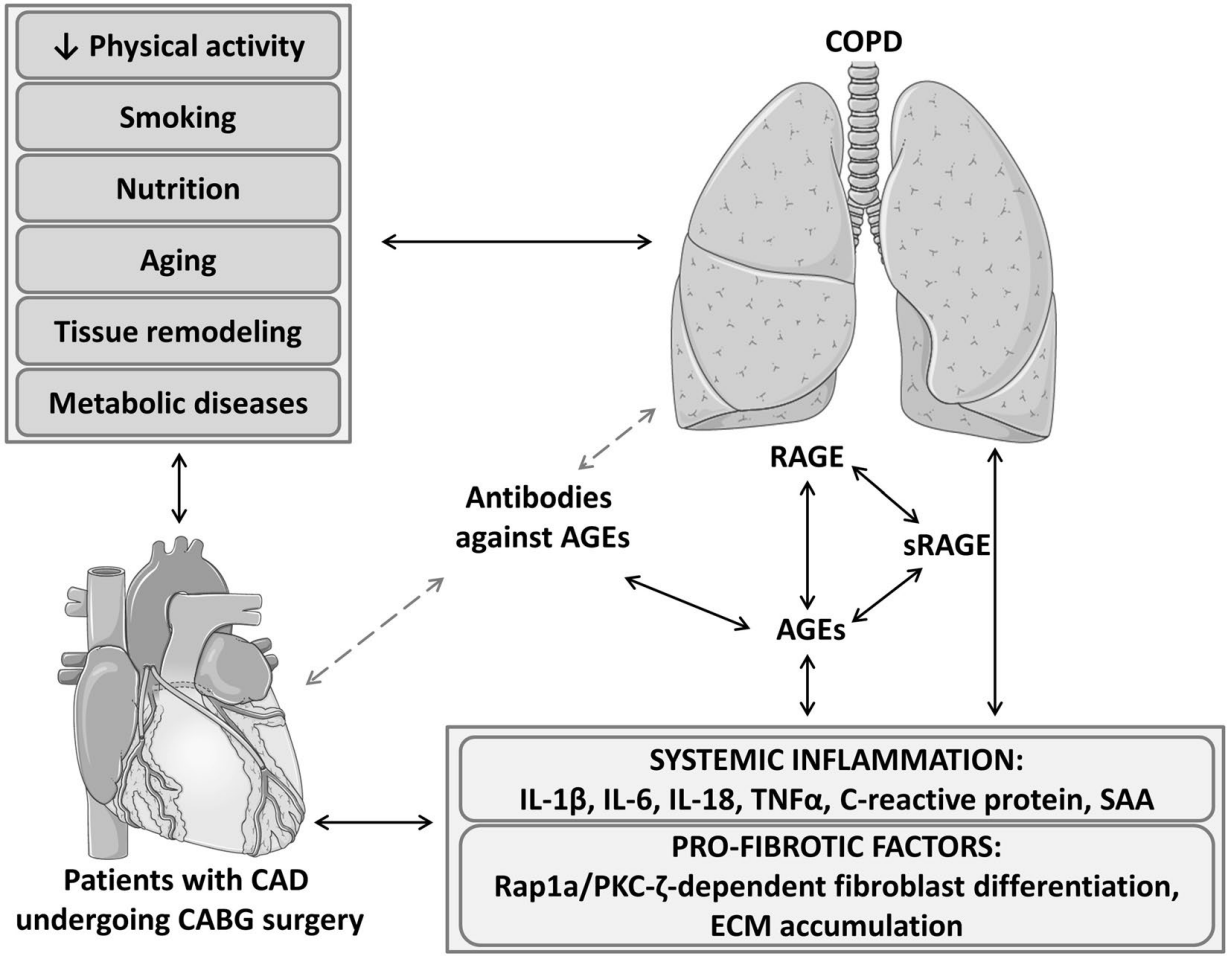
Molecular mechanisms, linking AGE-RAGE axis with systemic inflammation, and progression of COPD and CAD. CAD and COPD share a plethora of common risk factors, including lifestyle aspects and concomitant conditions, significantly exacerbating the course of both diseases. Chronic stimulation of the AGE-RAGE axis leads to overexpression of RAGE, completing the vicious circle that tunes metabolic and signaling pathways towards increased production of pro-fibrotic and pro-inflammatory factors. Anti-AGE-Abs might serve as an integral connector linking the risk factors of CAD and COPD with mechanisms triggering and supporting chronic inflammation, including AGE-RAGE-sRAGE triad. On the other hand by binding AGEs, anti-AGE-Abs might exert a protective function, similar to that of sRAGE. *AGEs* advanced glycation end-products, *CABG* coronary artery bypass grafting, *CAD* coronary artery disease, *ECM* extracellular matrix, *IL* interleukins, *PKC-ζ* protein kinase C isotype ζ, *RAGE* receptor to advanced glycation end-products, *Rap1a* small Ras-like GTPase, member of the Ras superfamily, *SAA* serum amyloid protein, *TNF* tumor necrosis factor.

In the present study we found a potential link between the amount of Anti-AGE-Abs and COPD in patients with CAD. COPD and CAD share many common risk factors^30^ (Fig. 2) and their combination has an adverse influence on the disease outcome compared to each disease alone^31^. AGE-RAGE axis signaling can be a potential point of interaction for the pathogenesis of both CAD and COPD. AGE/RAGE and Rap1a have independently been associated with increased myofibroblast formation and interstitial fibrosis. Zhao et al. proposed a potential role of Rap1a in the AGE/RAGE signaling pathway^32^. Rap1a, a small Ras-like GTPase can potentially activate PKC-ζ which in turn can phosphorylate Ser391 of the intracellular RAGE domain. RAGE phosphorylation at Ser391 is a ligand-dependent mechanism that is required to perpetuate AGE/RAGE signaling. Their preliminary results show that inhibition of Rap1a protein expression down-regulates the molecular switch used to activate PKC-ζ to promote AGE/RAGE-mediated fibrosis (Fig. 2). Evidence from animal research and clinical trials show an increased expression of RAGE and increase in RAGE-signaling in COPD^33^. Among other tissues, lung epithelium has the highest expression of RAGE. Cleaved circulating form of receptor, sRAGE is responsible for binding and eliminating AGE-modified proteins^34^. It is suggested that COPD progression is associated with declining levels of sRAGE^2^, potentially alleviating the natural AGE-clearance mechanism.

Whereas CAD and COPD have common causal factors, the exact mechanism linking these two has not yet been explained definitively. Both diseases are characterized by chronic systemic inflammation^34^. AGEs and Anti-AGE-Abs could represent a possible link between these two diseases (Fig. 2). Immune complexes consisting of AGEs and Anti-AGE-Abs might be an important mechanism through which a state of chronic inflammation is maintained. An interesting characteristic of COPD is that as the disease progresses the inflammation intensifies^35^. Research has shown that binding of AGEs to RAGE causes the activation of the proinflammatory pathway NF-κB and subsequent over-expression of the whole battery of cell adhesion molecules, cytokines, chemokines, acute phase reactants and RAGE^2^, creating a positive feedback loop, further intensifying the inflammatory process in COPD^2,21^.

We observed an increased amount of Anti-AGE-Ab in patients with COPD. These finding suggest an opposite role of sRAGE and Anti-AGE-Abs in COPD. While binding of AGEs to sRAGE may induce their clearance, binding of AGEs to autoantibodies may facilitate their deposition in tissues and lead to inflammation. Disease progression in COPD is accompanied by destruction of cells and the extracellular matrix in the lung. Wu et al. showed that the amount of AGEs on the surface of cells obtained from lung biopsies is higher in patients with COPD then in healthy probands^36^. Therefore, AGEs on the cell surface might serve as a target for antibodies which in turn mark the cells for destruction by the immune system.

We observed a possible, albeit week, link between BMI and anti-AGE-Ab levels in patients with CAD. Large body of evidence is accumulated on the role of AGEs in pathogenesis of obesity and metabolic disorders. Western diets, rich in ultra-processed foods, high in calories and added sugars are an established risk factor for unintended weight gain and obesity^14^. One part of the evidence addresses the causative role of dietary AGEs in chronic inflammation, oxidative stress, compromising endothelial function^23,37^. This mechanism was shown to be successfully mitigated with the help of dietary interventions, reducing the AGE-load and the amount of circulating AGEs^38^. On the other hand, obesity and metabolic syndrome are factors, instigating extensive endogenous production of AGEs^39^, undermining a further formation of autoantibodies against AGE-modified proteins.

Among patients with high levels of anti-AGE-Ab, enrolled in the current study, five were in need of postoperative dialysis. AGE-RAGE axis activation is described to have deleterious effects in patients with chronic kidney disease, inducing oxidative stress, exacerbating pathogenesis of cardiovascular complications and leading to secondary damage of the kidneys^40,41^. Due to the small number of patients in need of postoperative dialysis, results are inconclusive. For a better understanding of these findings further studies in large groups of patients are warranted.

Our study did not reveal any significant changes of anti-AGE-Ab kinetics in selected participants during the perioperative period up to one week after the operation. Time-points, selected for the measurements involved a very short time after the operation, when tissue damage could potentially induce the immune reaction towards tissue fragments, containing AGE-modified proteins. However, kinetics of IgM against AGEs might change and should be addressed separately in further research. In contrast to these results, earlier it was shown that levels of sRAGE change during and after the operation within the same time-scale^25^.

Discordance between AGE-autofluorescence and anti-AGE-Ab content of the plasma could be explained by high kinetic variability of circulating AGE levels. It is known that plasma concentrations of AGEs are fluctuating in response to dietary exposure^42^, activity of endogenous detoxifying enzymes^43^, gut microbiota^44^, among others. Anti-AGE-antibodies on the contrary have quite stable levels, rather providing an evidence of the long-term exposure to AGE-modified proteins. Plasma samples analyzed in this study were obtained from the patients before the operation in a fasted state, highly likely influencing AGEs and sRAGE content in plasma^45^.

Anti-AGE-Ab might play a protective role as well, serving as part of mechanism for removal of damaged or dysfunctional proteins. Engelbertsen et al. found that subjects with low levels of IgM recognizing MGO-modified p220 in apoB had an increased risk of cardiovascular events during 15 years follow-up^46^. Studies in animal models have shown that autoantibodies against carboxyethyllysine (CEL) enhance an uptake of CEL-modified albumin by macrophages^47^. In diabetic mice, immunization with AGE-modified BSA was shown to alleviate progression of diabetic nephropathy^48^. Physiological effects of anti-AGE autoantibodies and their potential protective properties in humans are under-investigated. Technologies employing humanized Anti-AGE-Ab as therapeutic agents for age-related diseases are being developed. Several patents, proposing Anti-AGE-Ab as a potential intervention for inflammatory, autoimmune, and neurodegenerative disorders were published^49,50^. To date, no products, based on this principle have entered the market or passed clinical trial successfully.

Initially, during the development stage of ELISA described in this study, the possibility of coating the plates with human serum albumin (HSA) instead of BSA was taken into consideration. However, immunological testing revealed that compared to BSA, HSA samples from different batches appeared to be more CML- and MG-H1-modified (Supplementary Fig. 3). HSA is usually derived from plasma pools of healthy adult donors, which explains the presence of AGE-modifications. Recombinant HSA, expressed in rice, was also highly AGE-modified. Ribose-modified BSA is a good model protein for detection of the Anti-AGE-Ab due to high prevalence of modifications (MG-H1, CML, pentosidine) (Supplementary Fig. 4) compared to BSA as a control due to low amount of modification and high structural similarity to native HSA.

Interestingly, variable amounts of IgGs against native BSA were identified in seven participants of the study Our experiments with immunodetection, using plasma pools from the above mentioned patients confirm that these antibodies are specifically binding to BSA and not HSA. Potential role of these antibodies remains elusive and debatable. Due to its presence in cow milk and beef, BSA is one of the earliest food proteins to which humans are exposed. In populations of patients with type 1 diabetes and celiac disease it was shown that levels of anti-BSA IgGs were higher, compared to healthy controls^51^. Studies confirm that BSA is immunogenic and can lead to production of IgE and IgG, not necessarily being an evidence of compromised intestinal barrier or autoimmune disease^26,52^. Most of the immunogenic epitopes of BSA reside in amino acid sequences that differ greatly from the ones in HSA^53^.

We have to address potential limitations of our study. The number of patients included in this study is low, therefore correlations or associations between anti-AGE antibody levels and clinical parameters are difficult to detect. The study population included patients of the older age with CAD, and did not include any properly matched control group of healthy individuals. Presence of comorbidity and multimorbidity (evident lipid and glucose metabolism disorders, high exposure to smoking), can possibly influence our data on plasma AGE-content and correspondingly, anti-AGE-Ab levels.

## Conclusion

In this study, high levels of anti-AGE-Ab in CAD patients correlated with COPD and higher BMI. Further research in diverse patient populations is warranted for better understanding of whether anti-AGE-Ab can serve as potential biomarkers of certain diseases, or allow better outcome prediction. Additional research should clarify whether these antibodies, besides AGEs by themselves or sRAGE as a binding partner of AGEs, may have a role in the pathophysiology of diseases.

## Supplementary information

Supplementary Information 1.

Supplementary Information 2.

Supplementary Information 3.

Supplementary Information 4.

## Data Availability

All data generated or analysed during this study are included in this published article (and its Supplementary Information files).
