# Supplementary material for: Circulating antibodies against age-modified proteins in patients with coronary atherosclerosis
Source: Sci Rep. 2020 Oct 13;10:17105. doi: 10.1038/s41598-020-73877-5 (PMC7553914; doi:10.1038/s41598-020-73877-5)
Supplement: Supplementary file 1 — Supplementary Information 1. [file 41598_2020_73877_MOESM1_ESM.docx]

**CIRCULATING ANTIBODIES AGAINST AGE-MODIFIED PROTEINS IN PATIENTS WITH CORONARY ATHEROSCLEROSIS**

**Edina Korça^1^, Veronika Piskovatska^1^, Jochen Börgermann^1, 2^, Alexander Navarrete Santos^3^, Andreas Simm^1, 3*^**

1 - Department of Cardiothoracic Surgery, University Hospital Halle (Saale), Martin-Luther University Halle-Wittenberg, Halle, Germany

2 – Present address: Herzzentrum Duisburg, Germany

3 - Center for Medical Basic Research, Martin-Luther University Halle-Wittenberg, Halle, Germany

**Supplementary Figures:**


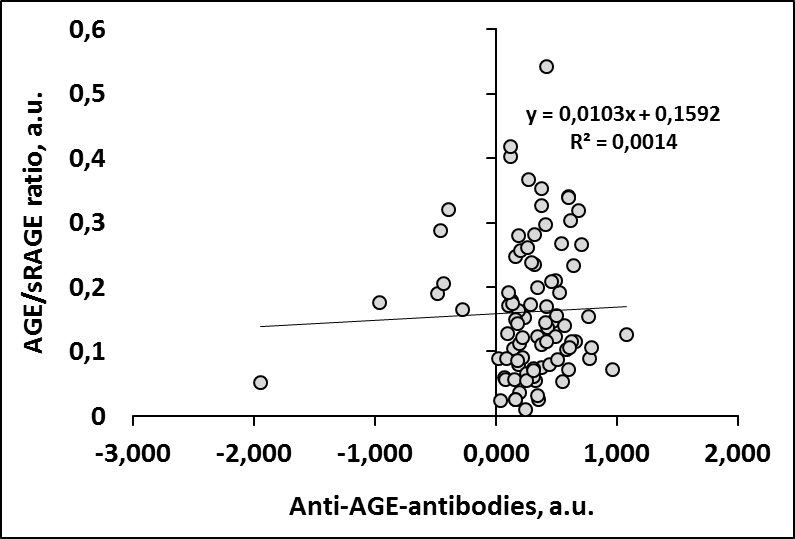


**Supplementary Figure 1. Ratio between AGE-index and sRAGE, plotted against levels of anti-AGE-antibodies.**

**
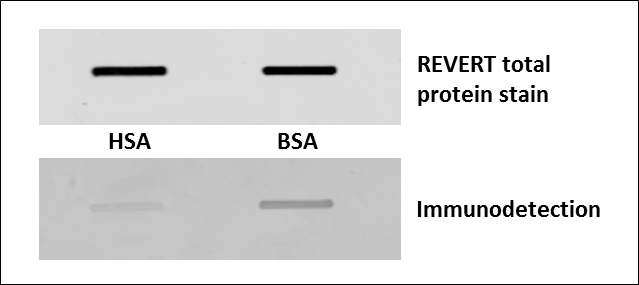
**

**Supplementary Figure 2. Specificity of anti-BSA antibodies in the plasma pool of patients with high anti-BSA reactivity.** Plasma pool was used as a source of anti-BSA antibodies (adapted from Mogues et al., 2005), HSA and BSA were blotted by slotblot, loading controlled by protein staining, treated with a plasma pool and IgGs against BSA detected with secondary anti-human IgG antibody (Li-Cor).

**
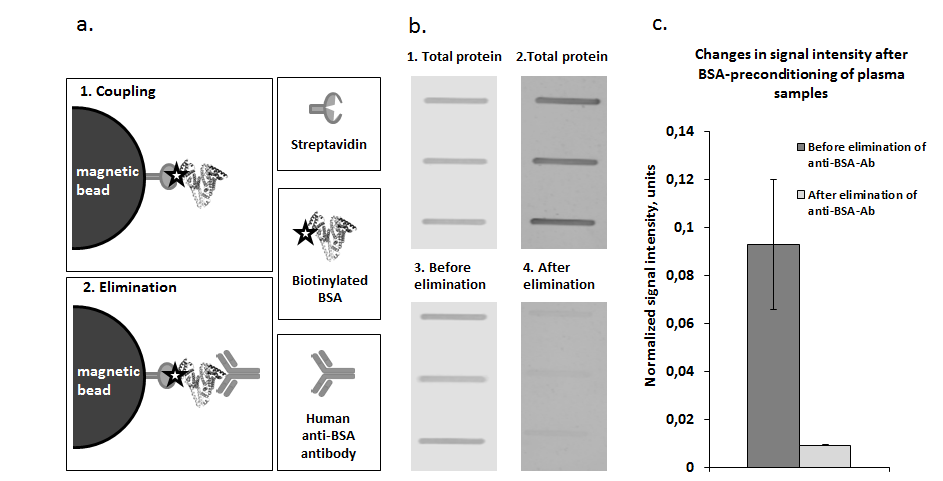
**

**Supplementary Figure 3. Experimental depletion of anti-BSA antibodies from human plasma.**

**A.** Elimination of anti-BSA antibodies from human plasma with streptavidin-magnetic beads, conjugated to biotinylated BSA: 1. Coupling of magnetic streptavidin beads with biotinylated BSA. 2. Elimination of anti-BSA antibodies from the plasma.

**B.** Representative slot-blot with immunodetection of anti-BSA antibodies in plasma pool from patients with high levels of anti-BSA-Ab before and after incubation with streptavidin magnetic beads- conjugated to biotinylated BSA. 1, 3. REVERT total protein stain. 2. Before elimination of anti-BSA-Ab. 4. After elimination of anti-BSA-Ab

**C.** Quantified results of the immunodetection. Changes in signal intensity before and after BSA-preconditioning of plasma samples mean ± SEM (n=3)


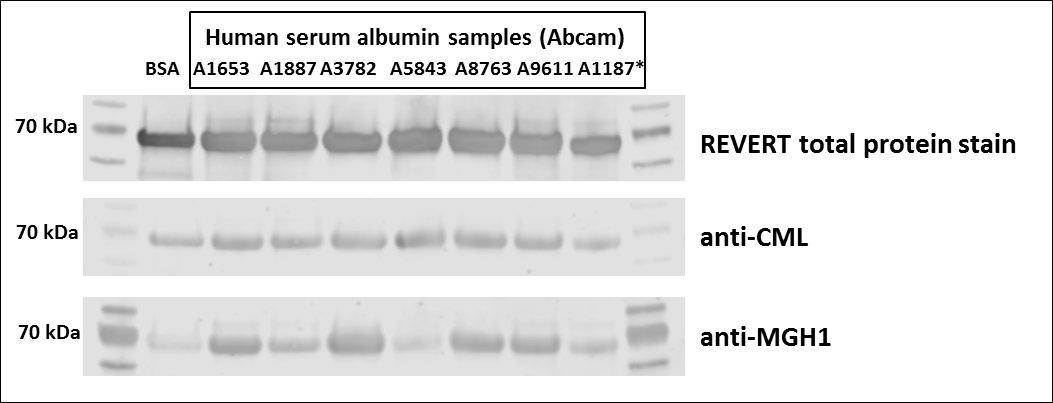


**Supplementary Figure 4.** **Detection of AGE-modifications in commercially available human serum albumin and BSA.** After normalization of signal from CML and MGH-1 towards total protein loading, BSA sample appeared to be the least modified in comparison to all human albumin samples. *two different batches of HSA A1887 were used for testing

**
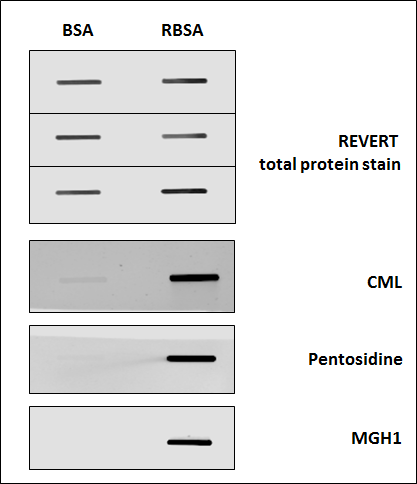
**

**Supplementary Figure 5.** **Detection of AGE-modifications in BSA and ribose modified BSA (RBSA).** After 40 days of incubation with reactive sugars, the RBSA sample had high amounts of CML, pentosidine and MGH-1, compared to non-modified BSA. Equal amounts of BSA and RBSA were blotted by slot-blot onto one membrane, stained for protein content (above) as a loading control, cut, washed, and analyzed the stripes for AGE modifications using anti-CML, Pentosidine or MGH1 antibodies.

Supplementary Figure 3 – original whole lane figures:

full-as-possible length original image: Supplementary Figure 3A: total protein stain

full-as-possible length original image: Supplementary Figure 3B: anti CML

full-as-possible length original image: Supplementary Figure 3C: anti MGH-1

**Figure legends:**

**Supplementary Figure 1. Ratio between AGE-index and sRAGE, plotted against levels of anti-AGE-antibodies.**

**Supplementary Figure 2. Specificity of anti-BSA antibodies in the plasma pool of patients with high anti-BSA reactivity.** Plasma pool was used as a source of anti-BSA antibodies (adapted from Mogues et al., 2005), HSA and BSA were blotted by slot blot, loading controlled by protein staining, treated with a patient plasma pool and IgGs against BSA detected with secondary anti-human IgG antibody (Li-Cor).

**Supplementary Figure 3. Experimental depletion of anti-BSA antibodies from human plasma.**

**A.** Elimination of anti-BSA antibodies from human plasma with streptavidin-magnetic beads, conjugated to biotinylated BSA: 1. Coupling of magnetic streptavidin beads with biotinylated BSA. 2. Elimination of anti-BSA antibodies from the plasma.

**B.** Representative slot-blot with immunodetection of anti-BSA antibodies in plasma pool from patients with high levels of anti-BSA-Ab before and after incubation with streptavidin magnetic beads- conjugated to biotinylated BSA. 1, 3. REVERT total protein stain. 2. Before elimination of anti-BSA-Ab. 4. After elimination of anti-BSA-Ab

**C.** Quantified results of the immunodetection. Changes in signal intensity before and after BSA-preconditioning of plasma samples mean ± SEM (n=3)

**Supplementary Figure 4. Detection of AGE-modifications in commercially available human serum albumin and BSA.** After normalization of signal from CML and MGH-1 towards total protein loading, BSA sample appeared to be the least modified in comparison to all human albumin samples. *two different batches of HSA A1887 were used for testing

**Supplementary Figure 5.** **Detection of AGE-modifications in BSA and ribose modified BSA (RBSA).** After 40 days of incubation with reactive sugars, the RBSA sample had high amounts of CML, pentosidine and MGH-1, compared to non-modified BSA. Equal amounts of BSA and RBSA were blotted by slot-blot onto one membrane, stained for protein content (above) as a loading control, cut, washed, and analyzed the stripes for AGE modifications using anti-CML, Pentosidine or MGH1 antibodies.
