# Supplementary material for: Circulating antibodies against age-modified proteins in patients with coronary atherosclerosis
Source: Sci Rep. 2020 Oct 13;10:17105. doi: 10.1038/s41598-020-73877-5 (PMC7553914; doi:10.1038/s41598-020-73877-5)
Supplement: Supplementary file 2 — Supplementary Information 2. [file 41598_2020_73877_MOESM2_ESM.pdf]

Image ID: 0001630_02
Acquire Time: 03.04.2019 10:10:33

Acquisition Information

#	Image ID	Acquire Time	Channels	Resolution	Intensities	Quality	Analysis	Image Name	Comment
1	0001630_02	03.04.2019 10:10:33	700 800	169um			Manual	0001630_02	

Image Display Values

Channel	Color	Minimum	Maximum	K
800	Gray Scale (Black on White)	0,455	4,95	0

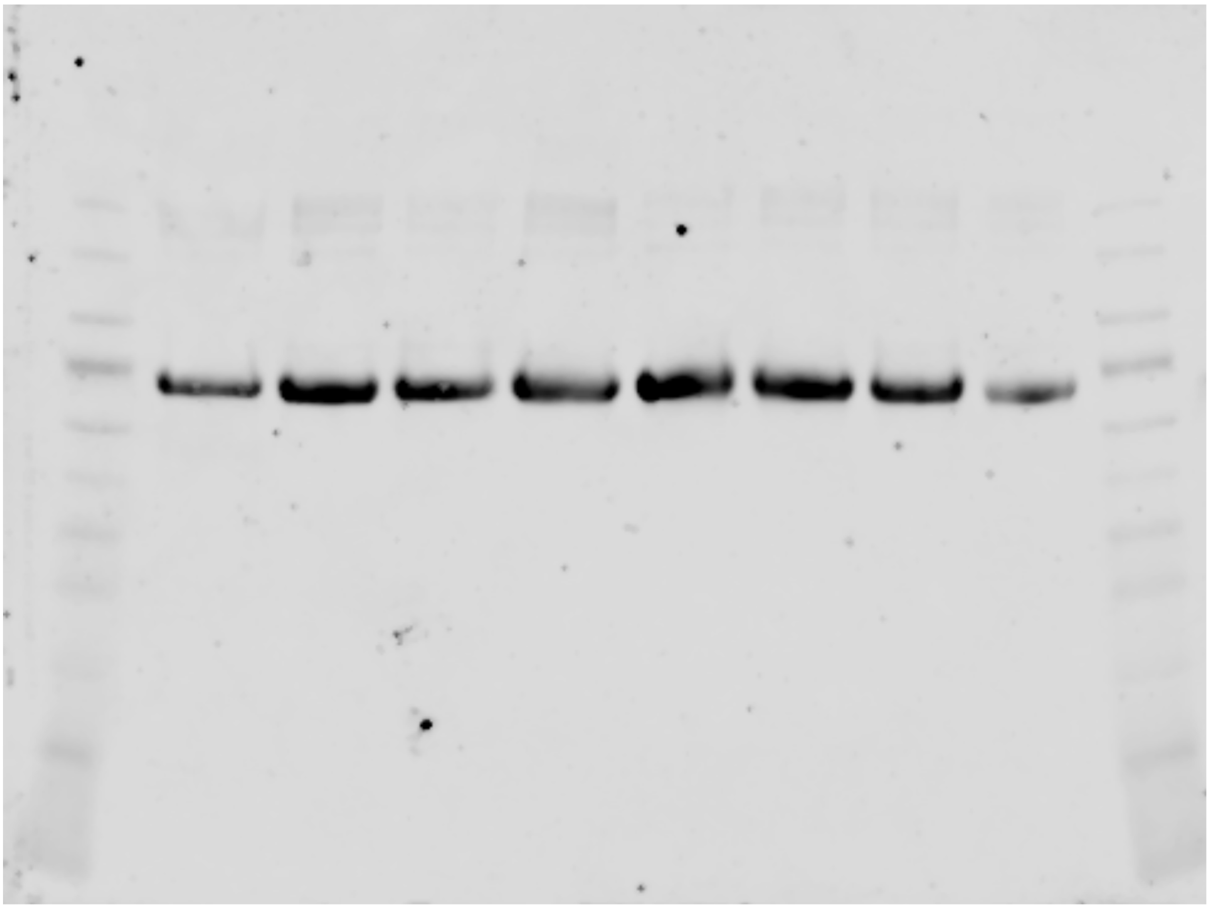

Acquisition Information (continued)

#	Image Modifications
1	
