# Supplementary material for: Circulating antibodies against age-modified proteins in patients with coronary atherosclerosis
Source: Sci Rep. 2020 Oct 13;10:17105. doi: 10.1038/s41598-020-73877-5 (PMC7553914; doi:10.1038/s41598-020-73877-5)
Supplement: Supplementary file 4 — Supplementary Information 4. [file 41598_2020_73877_MOESM4_ESM.pdf]

Image ID: 0001628_03
Acquire Time: 03.04.2019 10:10:31

Acquisition Information

#	Image ID	Acquire Time	Channels	Resolution	Intensities	Quality	Analysis	Image Name	Comment
1	0001628_03	03.04.2019 10:10:31	700	169um			Manual	0001628_03	

Image Display Values

Channel	Color	Minimum	Maximum	K
700	Gray Scale (Black on White)	46,9	614	0

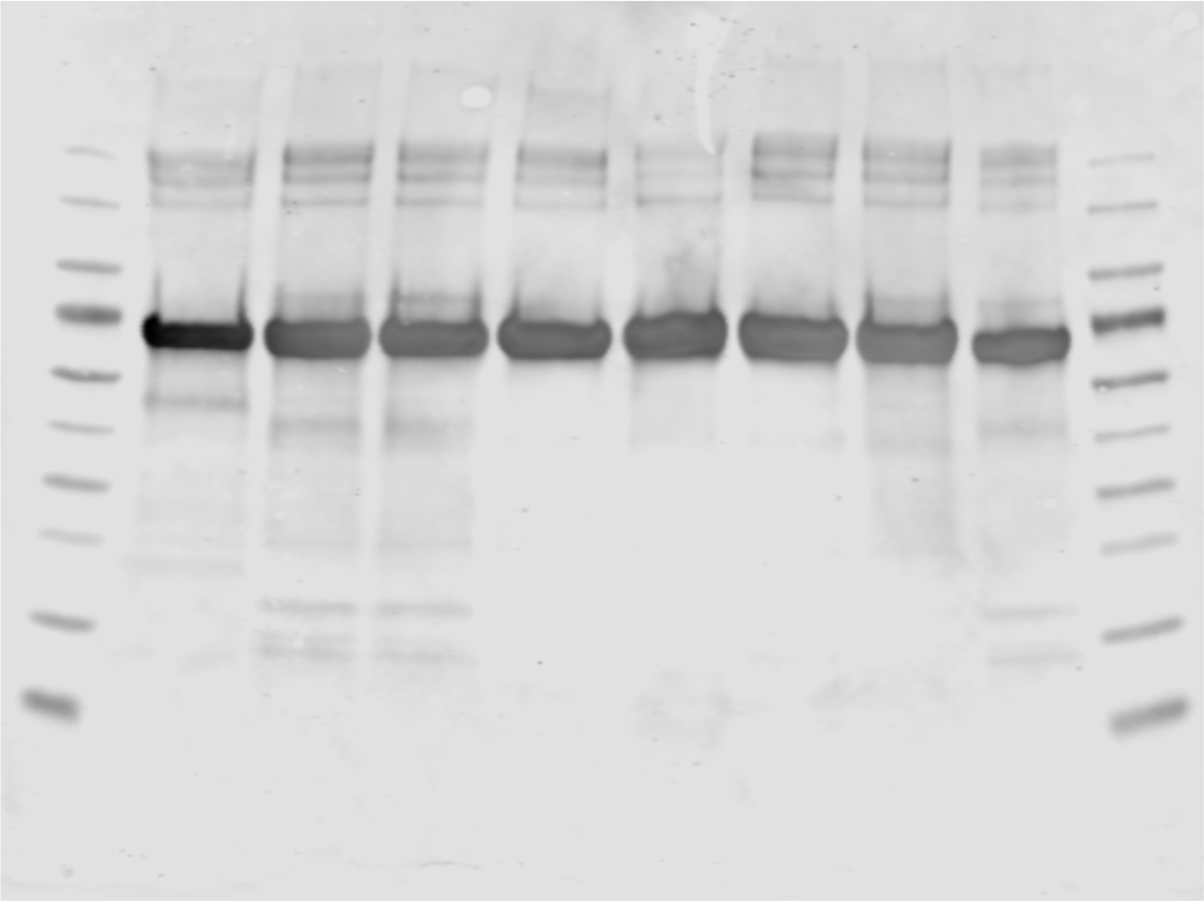

Acquisition Information (continued)

#	Image Modifications
1	
